# The miR-17-92 cluster as a potential biomarker for the early diagnosis of gastric cancer: evidence and literature review

**DOI:** 10.18632/oncotarget.15023

**Published:** 2017-02-02

**Authors:** Hong Li, Qiong Wu, Ting Li, Changhao Liu, Lin Xue, Jie Ding, Yongquan Shi, Daiming Fan

**Affiliations:** ^1^ State Key Laboratory of Cancer Biology and Xijing Hospital of Digestive Diseases, Xijing Hospital, Fourth Military Medical University, Xi'an 710032, China

**Keywords:** miR-17-92, circulating miRNA, intestinal metaplasia, gastric cancer, early diagnosis

## Abstract

**Purpose:**

Intestinal metaplasia is considered to be a pre-cancerous lesion of gastric cancer. The miR-17-92 cluster was previously reported to have clinical value in the prediction of cancer development. This study aimed to test the diagnostic value of miR-17-92 in gastric cancer and the intestinal metaplasia patients compared with the normal ones.

**Results:**

The results showed that miR-17-92 members were over-expressed in the serum of both gastric cancer and intestinal metaplasia patients, compared with healthy controls. Serum miR-17-92 members could also distinguish patients with gastric cancer and intestinal metaplasia from healthy controls.

**Materials and Methods:**

Serum miR-17-92 expression levels were detected using quantitative real-time PCR in 75 patients with gastric cancer, 104 patients with intestinal metaplasia and 38 healthy controls. The Receiver operating characteristic (ROC) curves and the area under the ROC curve (AUC) were then analyzed to test the efficacy of the miR-17-92 members in distinguishing gastric cancer, intestinal metaplasia and healthy controls.

**Conclusions:**

In conclusion, the miR-17-92 cluster might be useful as a potential serum biomarker for the early detection of gastric cancer.

## INTRODUCTION

Gastric cancer is one of the most common cancers with high mortality worldwide, and it is the most common cancer in East Asia. It is usually diagnosed at the late stages, and thus, it is important and imperative to make early diagnosis to try and reduce the high mortality rate of gastric cancer. Intestinal metaplasia is considered to be one of the most common pre-cancerous lesions of gastric cancer [1]. Therefore, the early diagnosis of intestinal metaplasia is also a key point in the early prevention of gastric cancer.

MiRNAs (microRNA) are non-coding RNAs of small molecular size that can regulate target gene expression by binding to their 3′ untranslated region [2]. miRNAs have been found to regulate various functions during cancer development, including cancer cell growth, metastasis, cell cycle, apoptosis, invasion, and chemo-resistance. The differential expression of miRNAs has been shown to be predictive of the occurrence or prognosis of cancers. miRNAs can be detected in cell-free body fluids, such as serum or plasma, and these miRNAs are called circulating miRNAs [3]. Circulating miRNAs are stable and more easily detected because they are protected from degradation by ribonucleases. According to these features, circulating miRNAs have been hypothesized and subsequently shown to be potential biomarkers of cancers. However, reports on the expression of miRNAs are often inconsistent, which makes it difficult to select a suitable miRNA as a useful biomarker [4].

The human miR-17-92 cluster, which is also named human oncomiR-1, is located on 13q31.3 and is a frequently amplified locus in cancers. The miR-17-92 cluster has seven members: miR-17-5p, miR-17-3p, miR-20a, miR-18a, miR-92a, miR-19a, and miR-19b. These miRNAs were previously identified to be over-expressed in tissue samples from various cancers, such as lung cancer [5], breast cancer [6], colon cancer [7], pancreatic cancer [8] and gastric cancer [9]. Additionally, the over-expressed miR-17-92 cluster members have been reported to have predictive value in the development of cancers and might have future clinical utility. For example, the up-regulation of the miR-17-92 cluster has been associated with tumor progression and prognosis in osteosarcoma and, thus, miR-17-92 might serve as a promising marker for tumor recurrence and survival in osteosarcoma patients [10]. Using Kaplan-Meier curves, Yu et al demonstrated a significant reduction in overall survival in patients expressing high levels of miR-17. Furthermore, Yu et al demonstrated a significant reduction in overall survival in patients expressing high levels of miR-17 [11]. The expression of the miR-17-92 members, miR-19a/b, was significantly associated with metastasis in gastric cancer patients [12]. The expression levels of miR-20a and miR-92a were also significantly associated with the overall survival of gastric cancer patients. Furthermore, a multivariate analysis revealed that miR-92a was an independent predictor of overall survival in gastric cancer patients [13].

Considering the reported utility of the miR-17-92 members in the prediction of cancer development and the increasing popularity of using circulating miRNAs as predictive tools for cancer diagnosis and prognosis, we assessed the relationship between circulating miR-17-92 levels and gastric cancer. By testing the levels of circulating miR-17-92 cluster members in gastric cancer patients, intestinal metaplasia patients and healthy controls, the present study focused on the clinical value of the expression of miR-17-92 members in the early diagnosis of gastric cancer.

## RESULTS

### Evaluation the possibility of using real-time PCR in measuring the miR-17-92 cluster levels in plasma samples

**Figure 1:**
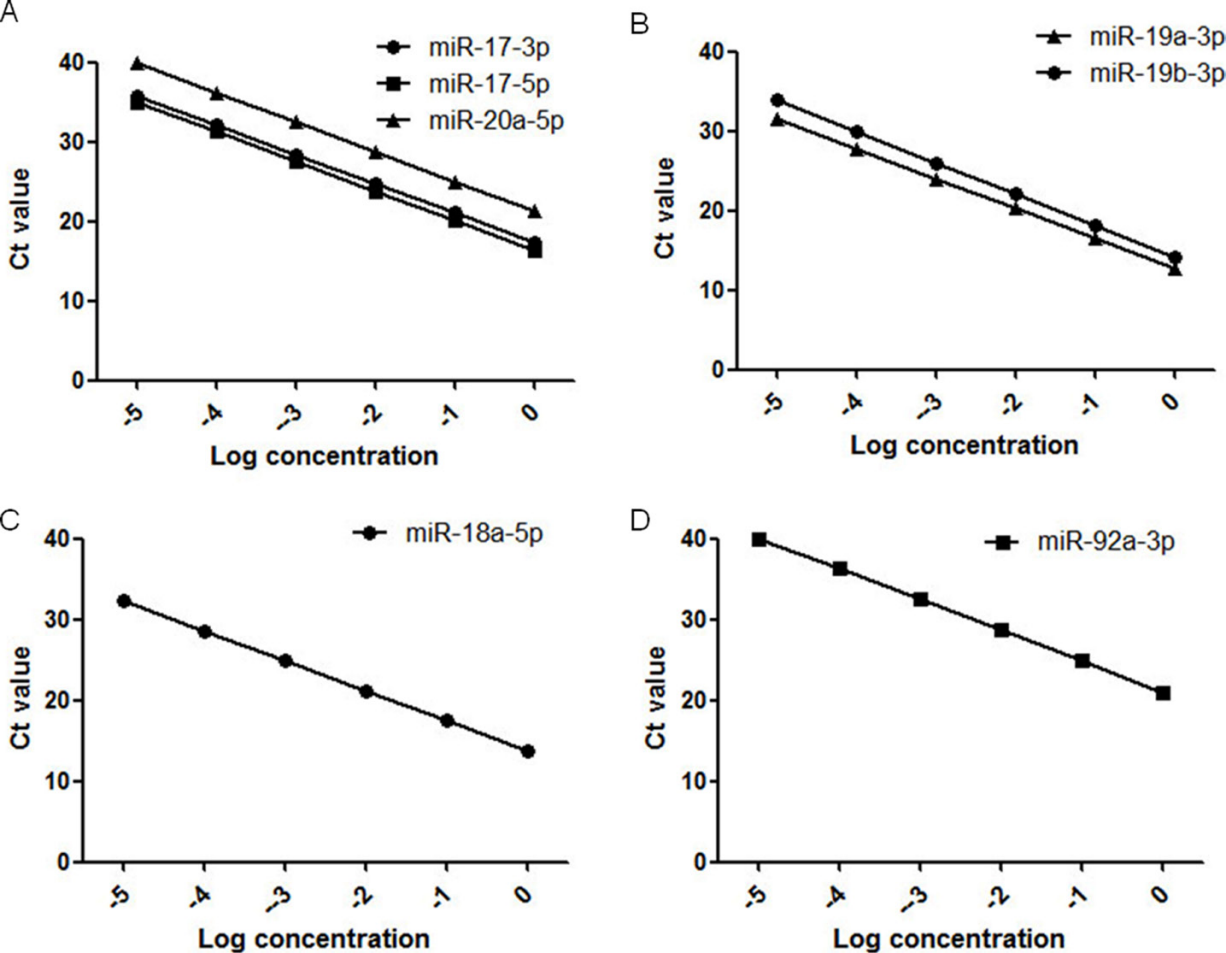
Ten-fold serial dilution of synthetic miR-17-92 miRNAs was used to generate the standard curves Linearity was confirmed within these concentrations, ranging from 1 to 0.00001 fmol. (**A**) Standard curves for miR-17-3p, miR-17-5p, and miR-20a-5p (**B**) Standard curves for miR-19a-3p and miR-19b-3p (**C**) Standard curve for miR-18a-5p (**D**) Standard curve for miR-92a-3p.

Considering that circulating miRNAs could be used as diagnostic markers, we thus wondered whether circulating miR-17-92 members would have diagnostic or predictive value in gastric cancer. Therefore, to evaluate the appropriateness of the serum assay, we first performed amplification for standard curve using real-time PCR for 10-fold serial dilutions of the miRNA reference panel. As shown in Figure 1, the relative coefficients, R^2^, were 0.999 (miR-17-3p), 0.998 (miR-17-5p), 0.998 (miR-18a-5p), 0.997 (miR-19a-3p), 0.998 (miR-19b-3p), 0.998 (miR-20a-5p), and 0.995 (miR-92a-3p). Using this method, we found that circulating miR-17-92 members could be amplified and detected in the serum samples that we collected from both patients and volunteers.

### Circulating miR-17-92 expression is higher in gastric cancer patients than healthy controls

**Figure 2:**
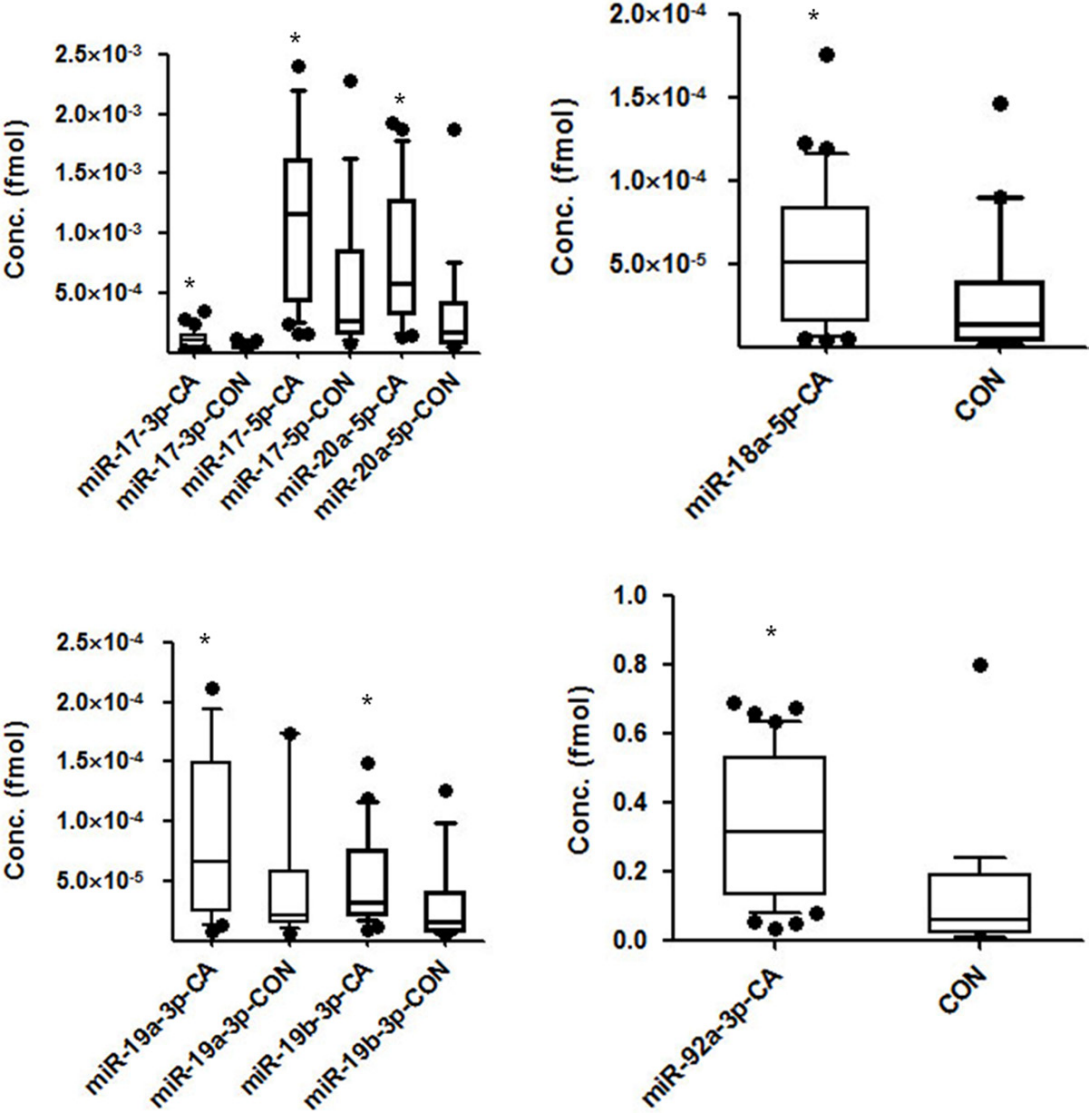
The expression levels of miR-17-92 cluster members in plasma from gastric cancer patients compared with that from healthy controls (*P < 0.05) (**A**) Expression levels of miR-17-3p, miR-17-5p, and miR-20a-5p (**B**) Expression levels of miR-19a-3p and miR-19b-3p (**C**) Expression level of miR-18a-5p (**D**) Expression level of miR-92a-3p.

miR-17-92 miRNA expression levels were previously identified to be highly expressed in gastric cancer patients’ gastrectomy samples, compared with the adjacent normal tissues [15, 16]. To assess the miR-17-92 miRNA expression patterns in the circulating serum of gastric cancer patients compared with healthy controls, and the exact expression results are listed in Supplementary Table 2. we performed real-time PCR using the serum samples from 75 gastric cancer patients and 38 healthy controls, and the exact expression results are listed in Supplementary Table 2. The expression concentrations of the miR-17-92 cluster members were significantly higher in the serums of gastric cancer patients compared with those of healthy controls, as indicated in Figure 2 (*p* < 0.05). Specifically, the expression level of miR-92a was the most significantly elevated.

### The combination of miR-17-3p and miR-19a-3p has higher predictive value for the diagnosis of gastric cancer

**Figure 3:**
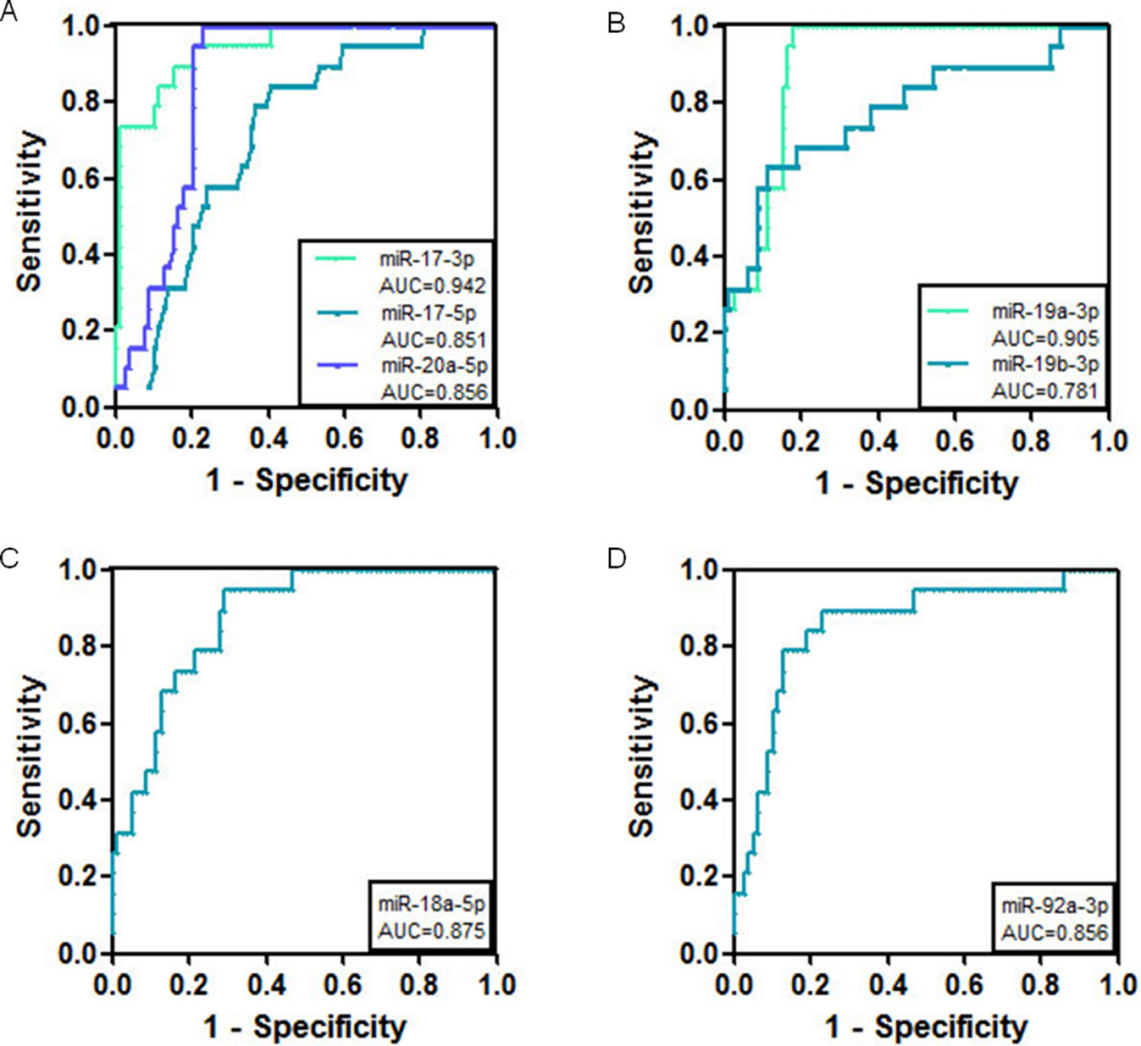
ROC curve analysis using serum miR-17-92 miRNAs for the discrimination of gastric cancer patients from volunteers (**A**) ROC curves for miR-17-3p, miR-17-5p, and miR-20a-5p (**B**) ROC curves for miR-19a-3p and miR-19b-3p (**C**) ROC curve for miR-18a-5p (**D**) ROC curve for miR-92a-3p.

**Figure 4:**
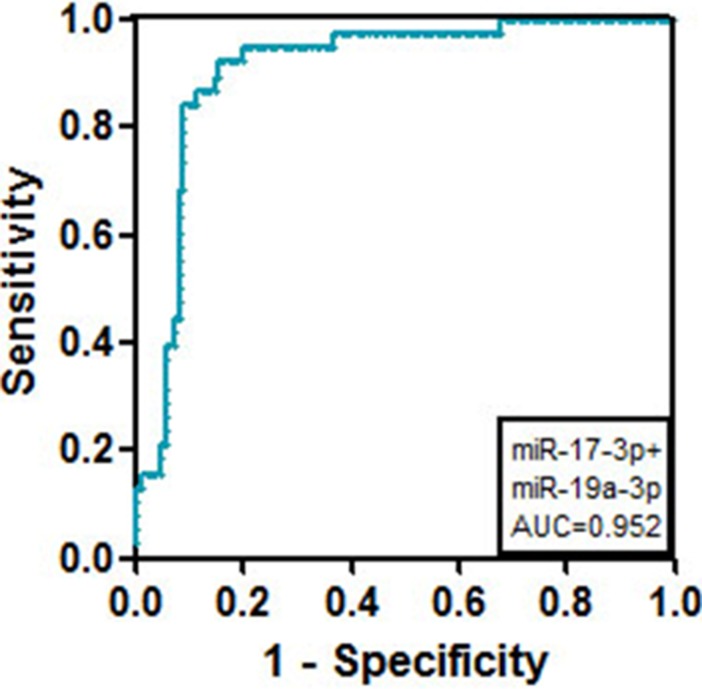
ROC curve analysis using the combination of serum miR-17-3p and miR-19a-3p for the discrimination of gastric cancer patients from volunteers

ROC curves for the plasma miRNAs were drawn using Graphpad Prism 5 software (GraphPad Software Inc, San Diego, California, USA), with the sensitivity as the y axis and the 1-specificity as the x axis. As shown in Figure 3 and Table 1, the AUC of miR-17-3p was the highest among the cluster members (AUC = 0.942 ± 0.02). Additionally, compared with healthy controls, single miR-17-3p had the highest specificity among the cluster members (Table 1). Except for miR-17-3p, the other members such as miR-17-5p, miR-18-5p, miR-19a-3p, miR-19b-3p, miR-20a-5p, and miR-92a-3p also showed diagnostic value for gastric cancer (Figure 3 and Table 1). The combined detection of miR-17-3p and miR-19a-3p showed a much higher specificity for the diagnosis of gastric cancer (94.7%), with a sensitivity of 63.16% and AUC of 0.952 (Figure 4 and Table 2).

**Table 1:** Comparison of the cut off values, sensitivity and specificity of miRNAs in gastric cancer serum vs those in healthy controls

MiRNAs	AUC	Cut off value (fmol)	Sensitivity (%)	Specificity (%)
miR-17-3p	0.942 ± 0.02	4.76E-3	70	89
miR-17-5p	0.851 ± 0.04	4.35E-4	65	75
miR-18a-5p	0.875 ± 0.03	4.81E-4	65	70
miR-19a-3p	0.905 ± 0.02	1.80E-3	74	87
miR-19b-3p	0.781 ± 0.06	6.68E-4	63	85
miR-20a-5p	0.856 ± 0.03	3.24E-4	64	79
miR-92a-3p	0.856 ± 0.05	2.84E-2	53	84

**Table 2:** Evaluation indicators of the joint detection of miR-17-3p and miR-19a-3p for discriminating GC patients

Evaluation indicators	miR17-3p+miR-19a-3p
AUC	0.952 ± 0.04
Cut off value (fmol)	3.45E-3
Sensitivity (%)	63.16
Specificity (%)	94.7

### Circulating miR-17-92 levels in intestinal metaplasia patients were even higher than in healthy controls

**Figure 5:**
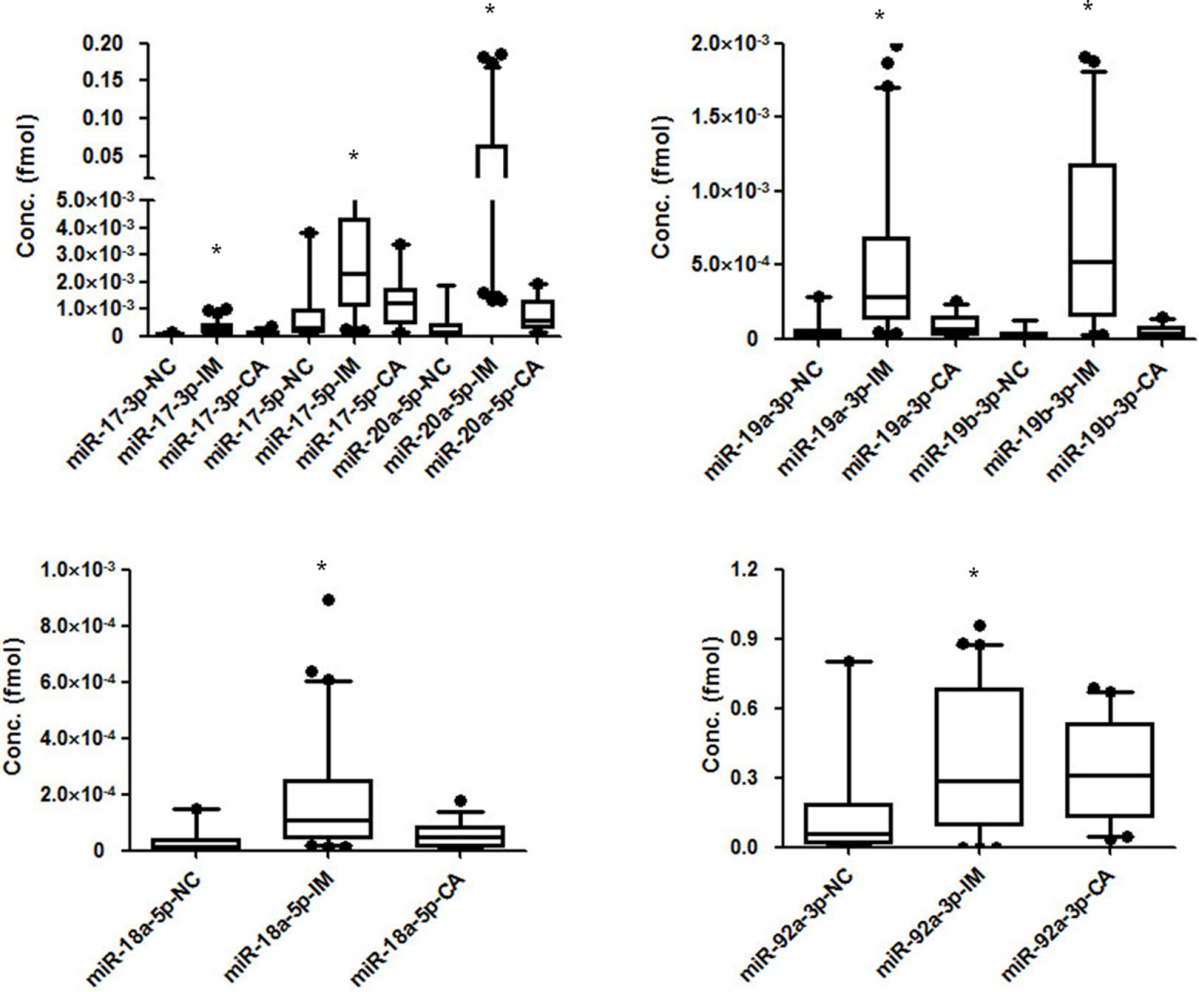
The expression levels of miR-17-92 cluster members in plasma from intestinal metaplasia patients compared with that from both gastric cancer patients and healthy controls (*P < 0.05) (**A**) Expression levels of miR-17-3p, miR-17-5p, and miR-20a-5p (**B**) Expression levels of miR-19a-3p and miR-19b-3p (**C**) Expression level of miR-18a-5p (**D**) Expression level of miR-92a-3p.

The expression levels of the miR-17-92 cluster members in the serums of 104 patients with intestinal metaplasia were examined using real-time PCR, the results are listed in Supplementary Table 2. As expected, the expression of the miR-17-92 members was significantly higher in intestinal metaplasia patients than in healthy controls. Interestingly, the expression levels of miR-17-92 members were also higher in those patients even compared to the gastric cancer patients as shown in Figure 5 (*p* < 0.05). The extremely higher expression of the miR-17-92 cluster in intestinal metaplasia patients might indicate special and complex functions of these miRNAs in the development of intestinal metaplasia and gastric cancer.

### Circulating miR-17-92 has higher diagnostic value in the detection of intestinal metaplasia

**Figure 6:**
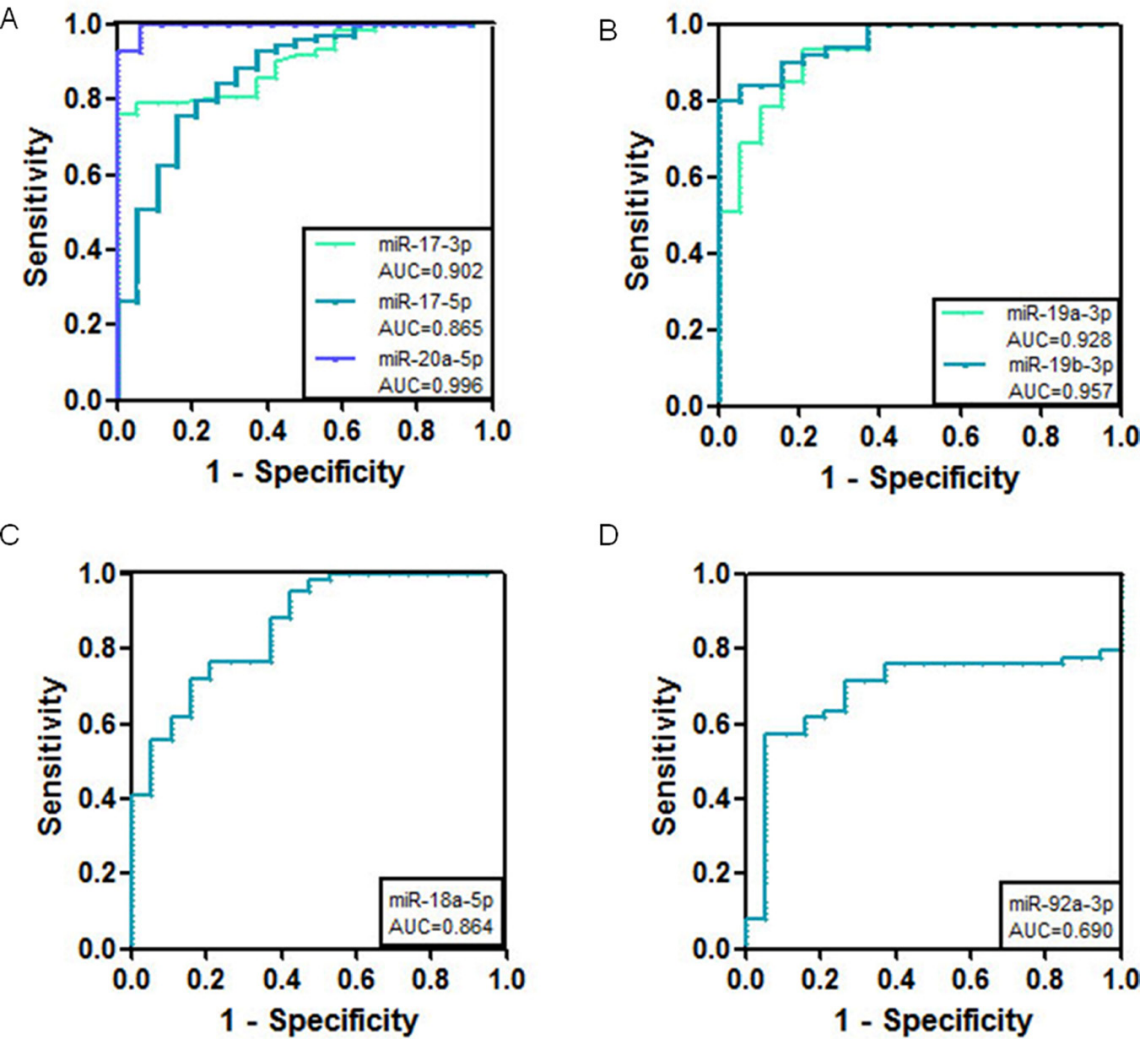
ROC curve analysis using serum miR-17-92 miRNAs for the discrimination of intestinal metaplasia from normal controls (**A**) ROC curves for miR-17-3p, miR-17-5p, and miR-20a-5p (**B**) ROC curves for miR-19a-3p and miR-19b-3p (**C**) ROC curve for miR-18a-5p (**D**) ROC curve for miR-92a-3p.

**Figure 7:**
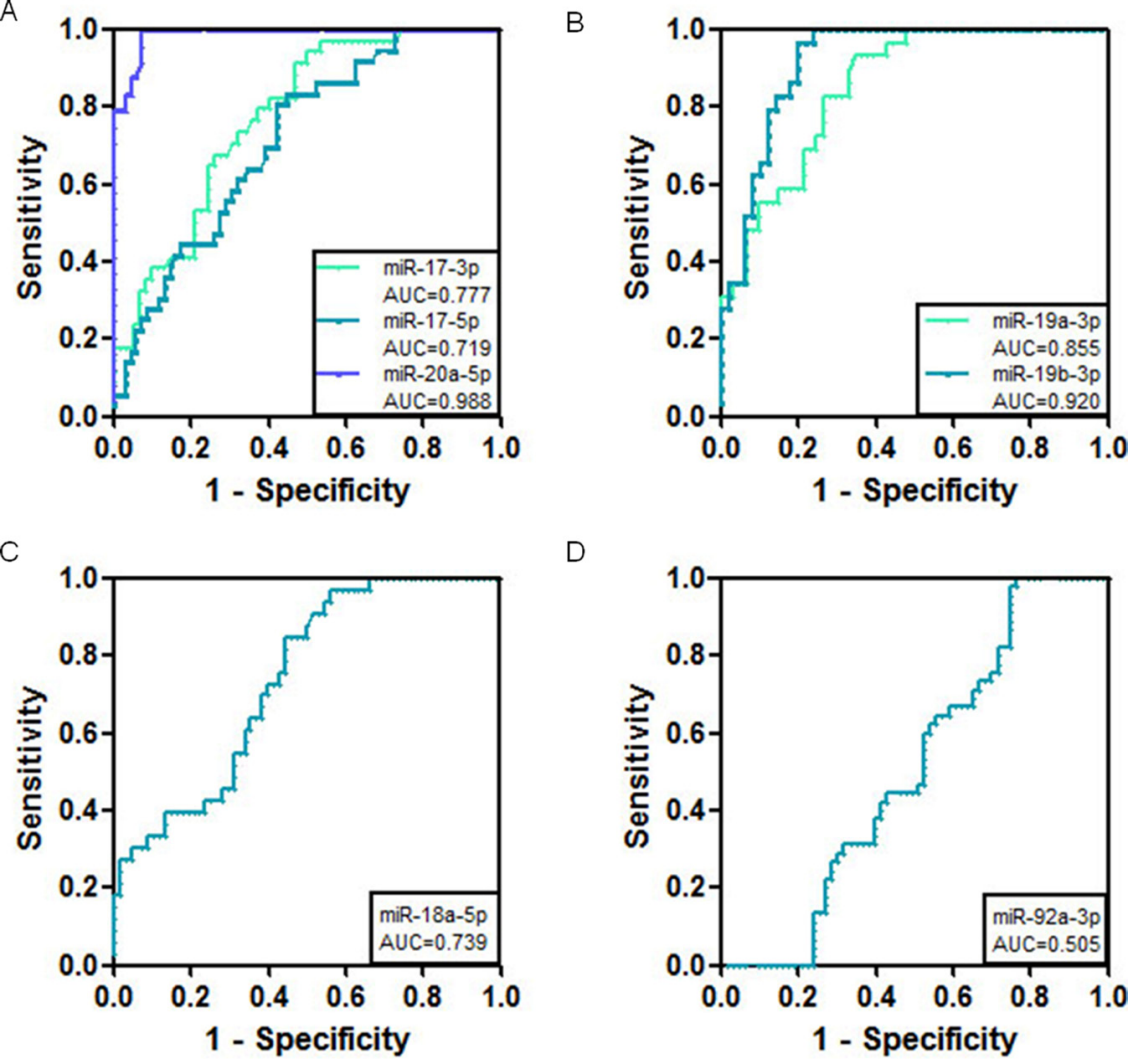
ROC curve analysis using serum miR-17-92 miRNAs for the discrimination of intestinal metaplasia from gastric cancer (**A**) ROC curves for miR-17-3p, miR-17-5p, and miR-20a-5p (**B**) ROC curves for miR-19a-3p and miR-19b-3p (**C**) ROC curve for miR-18a-5p (**D**) ROC curve for miR-92a-3p.

To test the early predictive value of miR-17-92 in the diagnosis of intestinal metaplasia, we further performed real-time PCR on serum samples from intestinal metaplasia patients to compare their miR-17-92 levels with those of healthy controls. In this study, 104 patients with intestinal metaplasia and 38 healthy controls were included. Interestingly, the results showed that the expression of all the miR-17-92 members had diagnostic values for distinguishing intestinal metaplasia patients from healthy controls or gastric cancer patients. Among them, miR-20a-5p had the largest AUC, with a sensitivity of 98% and a specificity of 95% for distinguishing intestinal metaplasia from healthy controls (Figure 6 and Table 3). When distinguishing intestinal metaplasia from gastric cancer, miR-20a also had the highest AUC, with a sensitivity of 93% and a specificity of 96%, as shown in Figure 7 and Table 4.

**Table 3:** Comparison of the cut off values, sensitivity and specificity of miRNAs in intestinal metaplasia serum vs those in healthy controls

MiRNAs	AUC	Cut off value (fmol)	Sensitivity (%)	Specificity (%)
miR-17-3p	0.902 ± 0.03	1.08E-3	79	94
miR-17-5p	0.865 ± 0.04	1.14E-2	75	84
miR-18a-5p	0.864 ± 0.04	4.75E-4	72	84
miR-19a-3p	0.928 ± 0.03	1.10E-3	79	89
miR-19b-3p	0.957 ± 0.02	9.97E-5	84	95
miR-20a-5p	0.996 ± 0.004	1.62E-2	98	95
miR-92a-3p	0.690 ± 0.06	1.98E-1	62	84

**Table 4:** Comparison of the cut off values, sensitivity and specificity of miRNAs in gastric cancer serum vs those in intestinal metaplasia

MiRNAs	AUC	Cut off value (fmol)	Sensitivity (%)	Specificity (%)
miR-17-3p	0.777 ± 0.04	1.37E-3	68	74
miR-17-5p	0.719 ± 0.04	1.74E-3	58	81
miR-18a-5p	0.739 ± 0.05	7.85E-5	60	72
miR-19a-3p	0.855 ± 0.04	1.54E-4	74	83
miR-19b-3p	0.920 ± 0.03	1.16E-4	80	90
miR-20a-5p	0.988 ± 0.007	1.90E-3	93	96
miR-92a-3p	0.505 ± 0.05	3.44E-1	48	60

## DISCUSSION

In recent years, it has been frequently reported that human fluids contain stably expressed miRNAs, which have been discovered to be predictive of the development of cancer. miRNAs are of small molecular size and are more easily secreted by the primary tumor and released into blood. Currently, circulating miRNAs have been discovered to have diagnostic or prognostic value in various cancers. For example, in patients with pancreatic ductal adenocarcinoma and those without pancreatic disease, a marked difference in the profiles of pancreatic juice of four circulating miRNAs (miR-205, miR-210, miR-492, and miR-1427) was observed. Additionally, the enrichment of the four miRNAs in pancreatic juice was associated with decreased overall survival. The increased levels of miR-205 and miR-210 have also been associated with lymph node metastasis in patients [17]. To identify circulating biomarkers that are able to predict clinical outcome in triple negative breast cancers, serum miRNA expression and real-time PCR analyses were performed. As a result, a four-miRNA signature (miR-18b, miR-103, miR-107 and miR-652) that predicted tumor relapse and overall survival was identified. Furthermore, multivariate Cox regression models indicated that the risk score based on the four-miRNA signature was an independent prognostic classifier in patients with triple negative breast cancer [18]. miR-17-92 cluster members were previously identified by our lab to be highly expressed in gastric cancer cell lines and tissues, to regulate gastric cancer progression and development, and to have prognostic value for gastric cancer patients [12–14]. After confirming that Real-time PCR can be used to measure the expression of miR-17-92 cluster members serums, we assessed the levels of this cluster members in gastric cancer patients’ serums compared with the normal volunteers. And we found an elevated expression of this cluster especially miR-92a in gastric cancer patients’ serums. As circulating miRNAs are mainly released from the primary tissue sites, the expression pattern of circulating miRNAs in the plasma or serum from cancer patients is often consistent with the expression of the miRNAs in the cancer tissues. For example, miR-18a expression was previously found to be significantly higher in gastric cancer tissues than in normal gastric tissues, and plasma miR-18a concentrations were significantly higher in gastric cancer patients than in healthy controls [20].

The present study also found that the combination of miR-17-3p and miR-19a-3p expression has higher predictive value for the diagnosis of gastric cancer. Both miR-17-3p and miR-19a-3p were previously demonstrated to regulate cancer progression or have predictive value in the diagnosis of cancers. For example, miR-17-3p was found to increase tumor cell proliferation, colony formation, cell survival and invasion by targeting TIMP3 in prostate tumor [21]. In hepatocellular carcinoma, miR-17-3p was reported to modulate proliferation, migration, survival, morphogenesis and colony formation of HepG2 cells, and it inhibited endothelial tube formation by repressing the expression of vimentin and GalNT7 [22]. The high expression of miR-19a-3p in serum was significantly associated with poor patient survival in astrocytoma patients [23]. Serum expression of miR-19a-3p, together with miR-223-3p, miR-92a-3p and miR-422a as a four-miRNA panel, had a high diagnostic accuracy for colorectal adenocarcinoma [24]. To the best of our knowledge, this is the first study to report the combined value of miR-17-3p and miR-19a-3p in the evaluation of the development of cancer.

The miR-17-92 cluster comprises 7 members: miR-17-5p, miR-17-3p, miR-18a, miR-19a, miR-19b, miR-20a and miR-92a. Circulating miR-17-92 was previously reported to be elevated in the blood of patients with lung cancer, as compared with subjects at risk for developing lung cancer [25]. Furthermore, Otsuji E et al found that the expression of miR-18a was significantly higher in pancreatic cancer tissues, esophageal squamous cell carcinoma tissues and gastric cancer tissues compared with the normal tissues. The plasma levels of miR-18a were significantly higher in pancreatic cancer patients, esophageal squamous cell carcinoma patients and gastric cancer patients than in control volunteers. Additionally, in the three cancer types, the plasma concentrations of miR-18a were significantly lower in postoperative samples than in preoperative samples [20, 26, 27]. The same group further found that the concentration of miR-18a in plasma/serum of patients with cancers, such as esophageal, pancreatic, hepatocellular, colorectal and other types of cancers, was much higher than that of healthy controls [28]. Among the cluster members, both miR-17-3p and miR-92 were significantly elevated in the patients with colorectal cancer. The plasma levels of these markers were significantly reduced after surgery in 10 patients with colorectal cancer [29]. The serum levels of miR-155, miR-19a, miR-181b, and miR-24 were significantly more abundant in high-risk, early-stage breast cancer patients in comparison to the low-risk group and showed a decreasing trend upon therapy [30].

With the continued study of the miR-17-92 cluster, the expression of the members of this cluster has been found to be associated with clinicopathological statistics or have predictive value for the development of various cancers. For example, higher miR-18a expression in plasma was associated with shorter disease-free survival and disease-specific survival in patients with gastric cancer [31]. Another group aimed to identify circulating microRNAs as early biomarkers of docetaxel response in castration-resistant prostate cancer patients. Interestingly, those authors found that decreased post-docetaxel levels of miR-17 family were identified in non-responders to docetaxel and patients who had shorter survival [32]. Wang et al also found that the plasma concentrations of miR-17-5p/20a were significantly associated with the differentiation status and TNM stages of gastric cancer and that the high expression levels of miR-17-5p/20a were significantly correlated with poor overall survival [33]. Furthermore, the high expression of miR-19a-3p was also significantly associated with poor patient survival [23]. The expression level of miR-20a in serum was significantly higher in cervical cancer patients compared to healthy controls, and patients with lymph node metastasis (LNM) tended to have over-expressed miR-20a [34]. However, the level of miR-92a was significantly lower in the tissue and serum samples of breast cancer patients than in those of healthy controls, and the decreased levels of miR-92a were associated with tumor size and positive lymph node status [35]. The differences of miR-92a expression between the previous study in breast cancer and this study in gastric cancer indicate that the exact role of miR-92a has not been illucidated. The discrepancy might because breast cancer was originated from the conduit epithelium, while gastric cancer was from mucosal epithelium. In colon cancer which is also originated from mucosal epithelium, the miR-92a expresion patten has been reported to be the same as what we are describing in gastric cancer in the present study. For example, plasma miR-92a was shown to have significant diagnostic value for advanced neoplasia and has strong potential as a novel, noninvasive biomarker for the early detection of colorectal cancer [36].

The serum expression of miR-17-92 was also detected by other groups in other cancers or pre-cancerous lesions. For example, in colon, miR-19b was reported to be significantly up-regulated in IBD patients, whereas in patients with colonic polyps, miR-18a was significantly up-regulated. On the other hand, miR-17, miR-19a, miR-20a and miR-223 were significantly up-regulated in CRC patients [37]. It was also found that both CEA and miR-17-3p were highly expressed in the serum of colon cancer patients. CEA plus miR-17-3p detection significantly increased the sensitivity and specificity in the test to discriminate stage I/II colon cancer patients from healthy controls [38]. In colorectal cancer, Expression of miR-17, miR-21 and miR-92 were significantly higher in serum of patients with disease relapse. Thus serum miR-21, miR-17, and miR-92 expression in colorectal cancer patients who underwent radical surgery and adjuvant chemotherapy may have diagnostic value in the differentiation between recurred and non-recurred patients [39].

Intestinal metaplasia is widely accepted to be a pre-cancerous lesion of gastric cancer and is known to increase the risk of gastric cancer by as much as 6 fold. From the aspect of pathology, gastric intestinal metaplasia is characterized by the loss of normal gastric epithelium and the replacement of the intestinal type cells, including goblet cells, Paneth cells, and absorptive cells [40–42]. Intestinal metaplasia can be divided into complete and incomplete types, with the latter considered to be associated with a greater risk of progression to gastric cancer [43]. However, it is clinically and pathologically difficult to make the distinction between the two types of gastric intestinal metaplasia because of the focal findings and the coexistence of the two types. The present study found that circulating miR-17-92 levels in intestinal metaplasia patients were even higher than in healthy controls. As a high-risk factor for gastric cancer, intestinal metaplasia was previously reported to be regulated by miRNAs. For example, the over-expression of miR-296-5p significantly promoted gastric cancer cell proliferation and attenuated CDX1-induced anti-growth effects by regulating cell cycle distribution and apoptotic status. Specifically, CDX1 has been reported to play vital roles in gastric intestinal metaplasia, and the identified miR-296-5p-CDX1-ERK1/2 axis elucidates our the understanding of the progression from intestinal metaplasia to gastric cancer [44]. The ectopic expression of CDX2 is also known to be associated with the development of intestinal metaplasia in stomach and gastric carcinogenesis. The over-expressed miR-9 might target CDX2 via its binding site in the 3′-UTR, resulting in the promotion of cell proliferation in gastric cancer [45]. The down-regulation of miR-203 and up-regulation of miR-196a correlate with the progression from intestinal metaplasia to adenocarcinoma, compared to normal individuals [46]. Although the above studies reveal that the progression of intestinal metaplasia might be regulated by miRNAs, only a few reports have discussed the value of circulating miRNAs in the evaluation of intestinal metaplasia.

Circulating molecules are popularly used as biomarkers to predict inflammation or cancer happening, development or recurrence. For example, AFP is currently used as the biomarker to test the existence of hepatic inflammation or hepatocellular carcinoma. An over 10 times increase of AFP or a progressively increasing of APF compared to the test value are regarded to have a bigger possibility of hepatocellular carcinoma. The present study found that miR-17-92 levels are relatively higher in intestinal metaplasia patients than both in gastric cancer patients and the healthy controls. Because intestinal metaplasia is a kind of lesion before gastric cancer, there is a possibility that miR-17-92 levels increased a lot in serums of intestinal metaplasia patients, and when it is dropping from the highest level, it might be the occurrence of cancer. However, the exact use of these miRNAs as biomarkers in clinic should base on a larger sample and several stages of clinical trials. The current study only provides the fact that miR-17-92 might become potential markers for intestinal metaplasia and gastric cancer.

The weakness of this study is that we used relatively fewer healthy controls compared to the patients and also the whole samples should be enlarged in future studies. In terms of the interesting fact that the intestinal metaplasia group has an even higher expression level than the gastric cancer group, we are expecting to find the reasons in further reseaches. To the best of our knowledge, the present study is the first report regarding the relationship between serum miRNA levels and the progression of intestinal metaplasia.

## MATERIALS AND METHODS

### Patients and samples

Serum samples were collected from patients who were diagnosed as having gastric cancer prior to gastractomy or having intestinal metaplasia without malignant lesions. In total, 75 patients with gastric cancer, 104 patients with intestinal metaplasia and 38 healthy controls were enrolled in this study. Immediately after collection, cell-free nucleic acids were isolated from the blood samples using a spin method 3000 r/min for 15 minutes to prevent contamination by cellular nucleic acids. The samples were then preserved at −80°C.

### Ethics statement

For the analyzed serum specimens, all patients gave informed consent to use excess pathological specimens for research purposes. The hospital's Protection of Human Subjects Committee approved the protocols used in this study. The use of human serum was approved by the institutional review board of the Fourth Military Medical University and conformed to the Helsinki Declaration and to local legislation. The patients whose samples were used in the study signed informed consent forms.

### RNA extraction and quantitative real-time PCR

Total RNA from serums was extracted using TRIZOL (Invitrogen, Carlsbad, CA, USA) and miRNeasy mini kit (QIAGEN Translational Medicine Co., Ltd, Suzhou, China) according to the manufacturer's instructions, with treatment with RNase-free DNase. Reverse transcription performed according to the manufacturer's instructions. Real-time PCR was performed to determine the expression levels of each miRNA, using the exact sequences (U to T) of these miRNAs as the forward primers and the unique q-PCR primer from the cDNA Synthesis Kit as the reverse primer the forward primer sequences are listed in Supplementary Table 1. U6 was used as an internal control, and each plate contained one cDNA sample for each primer as a calibration sample. Standard curve was measured using 10-fold serial dilutions of the miRNA reference panel. Concentrations of 0.00001 to 1 fmol of each synthetic miRNA between the logarithm of the amount of input miRNAs and the Ct values confirmed the linearity of the quantitative RT-PCR assay. Absolute quantification was performed using Bulge-Loop^™^ miRNA qRT-PCR Primer Set (RiboBio Co., Ltd, Guangzhou, China) and Uni-miR qPCR Primer (TaKaRa Biotechnology (DALIAN), Co., Ltd, DaLian, Liaoning Province, China). The exact real-time PCR steps are as follows: according to the manufacturer's instructions, 6.4 ul DEPC water and 10 ul SYBR were mixed with 0.8 ul sense and anti-sense primers each. cDNA from each sample was diluted 10 times after reverse-transcriptase. 18 ul above mixture and 2 ul cDNA were added into the eighth tube, 4 tubes for each sample. The parameters used in Lightcycler 480 PCR are 95°C pre-denaturation for 5 min, 40 cycles of 95°C for 10 sec and 60°C for 30 sec. Ct values were calculated by analyzing the melting curves. All experiments were performed in triplicate.

### Statistical analysis

Continuous variables were compared with ANOVA tests. If the test of homogeneity of variances between the groups was significant, the Mann-Whitney *U* test and Kruskal-Wallis H test were adopted as appropriate. A Chi-square or Fisher's test was used for categorical variables. Two-tailed *p*-values < 0.05 were considered to be statistically significant (*P < 0.05; **P < 0.01). ROC curves and the AUC were used to assess the feasibility of using the concentration of circulating miRNA as a diagnostic tool for detection. Youden index was used for the evaluation of the optimal cut-off point. The ROC curves, the standard curves and the figures for expression levels were drawn using Graphpad Prism 5 software (GraphPad Software Inc, San Diego, California, USA). All statistical analyses were conducted using SPSS software, version 14.0 (Chicago, Illinois, USA).

## CONCLUSIONS

Although the miR-17-92 cluster members have previously been found to regulate the progression of cancers, few studies have focused on the utility of serum miR-17-92 values in the early diagnosis of cancers. The present study reveals that miR-17-92 cluster members not only have distinguishing values in gastric cancer, but also have predictive ability in identifying intestinal metaplasia, the pre-cancerous stage of gastric cancer. More importantly, the miR-17-92 can also distinguish the early stages of cancer, indicating that the expression of the miR-17-92 cluster might have significant roles in the early diagnosis of gastric cancer.

## SUPPLEMENTARY MATERIALS TABLES




