# Supplementary material for: The miR-17-92 cluster as a potential biomarker for the early diagnosis of gastric cancer: evidence and literature review
Source: Oncotarget. 2017 Feb 2;8(28):45060–71. doi: 10.18632/oncotarget.15023 (PMC5542167; doi:10.18632/oncotarget.15023)
Supplement: Supplementary file 1 [file oncotarget-08-45060-s001.pdf]

The miR-17-92 cluster as a potential biomarker for the early diagnosis of gastric cancer: evidence and literature review

Supplementary Materials

Supplementary Table 1: Primer sequences of miR-17-92 cluster used in real-time PCR analysis

miRNA	Primer sequence
miR-17-3p	ACTGCAGTGAAGGCACTTGTAG
	Unique q-PCR reverse primer from the cDNA Synthesis Kit
miR-17-5p	CAAAGTGCTTACAGTGCAGGTAG
	Unique q-PCR reverse primer from the cDNA Synthesis Kit
miR-18a-5p	TAAGGTGCATCTAGTGCAGATAG
	Unique q-PCR reverse primer from the cDNA Synthesis Kit
miR-19a-3p	TGTGCAAATCTATGCAAAACTGA
	Unique q-PCR reverse primer from the cDNA Synthesis Kit
miR-19b-3p	TGTGCAAATCCATGCAAAACTGA
	Unique q-PCR reverse primer from the cDNA Synthesis Kit
miR-20a-5p	TAAAGTGCTTATAGTGCAGGTAG
	Unique q-PCR reverse primer from the cDNA Synthesis Kit
miR-92a-3p	TATTGCACTTGTCCCGGCCTGT
	Unique q-PCR reverse primer from the cDNA Synthesis Kit

Supplementary Table 2: Intestinal metaplasia. See Supplementary_Table_2
