# Supplementary material for: The miR-17-92 cluster as a potential biomarker for the early diagnosis of gastric cancer: evidence and literature review
Source: Oncotarget. 2017 Feb 2;8(28):45060–71. doi: 10.18632/oncotarget.15023 (PMC5542167; doi:10.18632/oncotarget.15023)
Supplement: Supplementary file 2 [file oncotarget-08-45060-s002.docx]

| **Intestinal Metaplasia** | | | | | | | | | |
| --- | --- | --- | --- | --- | --- | --- | --- | --- | --- |
| Number | Gender | Age | miR-17-3p | miR-17-5p | miR-18a-5p | miR-19a-3p | miR-19b-3p | miR-20a-5p | miR-92a-3p |
| 1 | M | 50 | 0.000933 | 0.001662 | 0.003339 | 0.002733 | 0.001869 | 0.17329 | 0.540635 |
| 2 | M | 44 | 5.99E-05 | 0.001287 | 4.42E-05 | 8.4E-05 | 9.6E-05 | 0.012743 | 0.102978 |
| 3 | F | 51 | 8.24E-05 | 0.000309 | 3.04E-05 | 2.99E-05 | 2.73E-05 | 0.004627 | 0.000904 |
| 4 | F | 58 | 2.92E-05 | 0.000845 | 2.11E-05 | 3.32E-05 | 3.49E-05 | 0.007149 | 0.000405 |
| 5 | M | 54 | 0.00085 | 0.005011 | 0.000594 | 0.000412 | 0.001108 | 0.156921 | 0.011395 |
| 6 | M | 40 | 0.000391 | 0.002838 | 0.000576 | 0.00049 | 0.001082 | 0.056278 | 0.004858 |
| 7 | F | 66 | 0.000308 | 0.001026 | 0.000133 | 0.00079 | 0.001574 | 0.181034 | 0.003931 |
| 8 | M | 67 | 0.000158 | 0.000694 | 2.02E-05 | 4.52E-05 | 5.94E-05 | 0.006313 | 0.001997 |
| 9 | F | 54 | 0.00019 | 0.002718 | 0.000129 | 0.000205 | 0.000346 | 0.028408 | 0.002498 |
| 10 | M | 66 | 0.000148 | 0.002909 | 0.000242 | 0.000362 | 0.000552 | 0.085876 | 0.001903 |
| 11 | M | 61 | 6.06E-05 | 0.000516 | 4.34E-05 | 4.6E-05 | 4.84E-05 | 0.006845 | 0.000787 |
| 12 | F | 50 | 0.000293 | 0.004456 | 0.000361 | 0.00095 | 0.001224 | 0.061394 | 0.004176 |
| 13 | M | 69 | 3.82E-05 | 0.001069 | 1.94E-05 | 6.41E-05 | 9.11E-05 | 0.006045 | 0.000872 |
| 14 | M | 59 | 0.00013 | 0.005397 | 0.000189 | 0.000392 | 0.000415 | 0.029305 | 0.001646 |
| 15 | M | 43 | 0.000239 | 0.00348 | 0.000112 | 0.000596 | 0.001306 | 0.004656 | 0.69153 |
| 16 | M | 30 | 0.001 | 0.005848 | 0.000148 | 0.000867 | 0.001869 | 0.010641 | 0.104807 |
| 17 | M | 66 | 0.000515 | 0.002477 | 8.19E-05 | 0.00069 | 0.00168 | 0.008886 | 0.302024 |
| 18 | M | 58 | 0.034089 | 0.003272 | 0.000122 | 0.003559 | 0.00103 | 0.100311 | 0.254521 |
| 19 | M | 55 | 0.000125 | 0.001158 | 2.4E-05 | 0.000202 | 0.001376 | 0.001327 | 0.347089 |
| 20 | F | 60 | 0.001589 | 0.001774 | 4.91E-05 | 0.001612 | 0.001639 | 0.021081 | 0.194241 |
| 21 | M | 46 | 0.000133 | 0.002315 | 4.82E-05 | 0.00033 | 0.001302 | 0.002695 | 0.275836 |
| 22 | F | 32 | 0.000286 | 0.000916 | 2.05E-05 | 0.000166 | 0.000603 | 0.001343 | 0.151591 |
| 23 | M | 48 | 0.002493 | 0.006953 | 0.000232 | 0.008833 | 0.001921 | 0.125719 | 0.092212 |
| 24 | F | 57 | 0.000259 | 0.004267 | 0.000891 | 0.03228 | 0.001412 | 0.16492 | 0.042119 |
| 25 | F | 42 | 0.005802 | 0.001923 | 9.75E-05 | 0.007483 | 0.001231 | 0.052233 | 0.02843 |
| 26 | M | 58 | 0.000335 | 0.001202 | 5.57E-05 | 0.000388 | 0.001678 | 0.003089 | 0.045563 |
| 27 | F | 63 | 0.000118 | 0.001011 | 4.42E-05 | 0.000205 | 0.001039 | 0.001691 | 0.021268 |
| 28 | M | 56 | 0.00029 | 0.001319 | 5.36E-05 | 0.000246 | 0.001376 | 0.001589 | 0.037321 |
| 29 | F | 48 | 0.002079 | 0.029894 | 0.000167 | 0.002192 | 0.001234 | 0.097848 | 0.12884 |
| 30 | F | 42 | 0.001778 | 0.009588 | 0.000414 | 0.001715 | 0.00122 | 0.067392 | 0.101774 |
| 31 | M | 66 | 0.002079 | 0.004374 | 0.000165 | 0.001622 | 0.001013 | 0.017935 | 0.079953 |
| 32 | F | 57 | 0.000386 | 0.00136 | 2.91E-05 | 0.000203 | 0.000951 | 0.001659 | 0.180646 |
| 33 | M | 52 | 0.000566 | 0.006868 | 0.000535 | 0.002358 | 0.000908 | 0.143845 | 0.14995 |
| 34 | M | 66 | 0.000722 | 0.002181 | 0.000148 | 0.002287 | 0.000757 | 0.118638 | 0.068322 |
| 35 | M | 51 | 0.000414 | 0.005532 | 0.000271 | 0.001868 | 0.0005 | 0.065738 | 0.087544 |
| 36 | M | 39 | 0.001071 | 0.002668 | 0.000173 | 0.001178 | 0.00013 | 0.011975 | 0.428897 |
| 37 | F | 53 | 0.000286 | 0.000809 | 3.2E-05 | 0.000128 | 2.66E-05 | 0.006313 | 0.173159 |
| 38 | M | 66 | 5.35E-05 | 0.001377 | 3.06E-05 | 0.000167 | 0.000107 | 0.012664 | 0.158145 |
| 39 | M | 35 | 0.000182 | 0.000461 | 1.57E-05 | 0.000159 | 2.22E-05 | 0.005752 | 0.102324 |
| 40 | F | 45 | 0.000588 | 0.015818 | 0.000636 | 0.004024 | 0.000762 | 0.123145 | 0.717079 |
| 41 | F | 55 | 0.000337 | 0.002272 | 0.0002 | 0.000287 | 0.0001 | 0.039003 | 0.201417 |
| 42 | M | 38 | 0.008237 | 0.009828 | 0.000609 | 0.004306 | 0.001736 | 0.177139 | 0.121697 |
| 43 | F | 61 | 0.000326 | 0.003095 | 0.000346 | 9.04E-05 | 0.001983 | 0.048631 | 0.106671 |
| 44 | F | 56 | 0.000197 | 0.003272 | 0.000117 | 0.000674 | 0.000203 | 0.038045 | 0.073021 |
| 45 | F | 58 | 0.00031 | 0.005995 | 0.000466 | 0.000851 | 0.000453 | 0.104771 | 0.69153 |
| 46 | M | 62 | 0.000345 | 0.007966 | 0.000481 | 0.001986 | 0.001791 | 0.126779 | 0.060907 |
| 47 | M | 45 | 0.026375 | 0.001899 | 0.000218 | 0.000894 | 0.001232 | 0.017604 | 0.071708 |
| 48 | M | 37 | 0.000798 | 0.00152 | 0.000155 | 0.00055 | 0.0013 | 0.019688 | 0.386999 |
| 49 | M | 44 | 0.000297 | 0.001983 | 0.000104 | 0.0004 | 0.001876 | 0.034875 | 0.078043 |
| 50 | F | 46 | 0.000203 | 0.001492 | 0.000124 | 0.000425 | 0.001238 | 0.021081 | 0.362096 |
| 51 | M | 57 | 5.91E-05 | 0.000278 | 5.3E-05 | 6.77E-05 | 0.000471 | 0.006236 | 0.007738 |
| 52 | M | 49 | 0.001447 | 0.003212 | 0.000266 | 0.000392 | 0.001218 | 0.058054 | 0.880751 |
| 53 | M | 65 | 0.002879 | 0.00572 | 0.000481 | 0.001075 | 0.001257 | 0.173327 | 0.073463 |
| 54 | F | 53 | 0.000555 | 0.000213 | 1.94E-05 | 6.99E-05 | 0.000246 | 0.002501 | 0.094019 |
| 55 | F | 54 | 0.000301 | 0.00066 | 3.6E-05 | 0.00014 | 0.00074 | 0.011324 | 0.270878 |
| 56 | M | 60 | 0.00061 | 0.000194 | 1.64E-05 | 9.1E-05 | 0.000474 | 0.0362 | 0.183953 |
| 57 | F | 43 | 0.003263 | 0.008015 | 0.000359 | 0.002112 | 0.00796 | 0.122087 | 0.112175 |
| 58 | M | 68 | 0.004809 | 0.003153 | 0.00021 | 0.001276 | 0.001909 | 0.185591 | 0.408643 |
| 59 | M | 52 | 0.000352 | 0.004889 | 0.000253 | 0.000846 | 0.001337 | 0.04444 | 0.695724 |
| 60 | M | 56 | 0.000149 | 0.002358 | 9.81E-05 | 0.000245 | 0.001749 | 0.018501 | 0.121348 |
| 61 | F | 67 | 4.87E-05 | 0.00077 | 2.93E-05 | 0.000182 | 0.001009 | 0.012125 | 0.279192 |
| 62 | M | 46 | 0.000233 | 0.001611 | 0.000153 | 0.003975 | 0.001791 | 0.128621 | 0.200791 |
| 63 | M | 67 | 0.000245 | 0.001597 | 6.19E-05 | 0.000194 | 0.001161 | 0.011184 | 0.071708 |
| 64 | M | 45 | 3.54E-05 | 0.001005 | 3.22E-05 | 7.85E-05 | 0.00048 | 0.005896 | 0.398878 |
| 65 | M | 40 | 6.74E-05 | 0.001627 | 5.67E-05 | 0.000198 | 0.001275 | 0.012276 | 0.060907 |
| 66 | M | 45 | 0.000687 | 0.0067 | 0.000299 | 0.001714 | 0.001742 | 0.132469 | 0.069994 |
| 67 | F | 68 | 0.000163 | 0.005074 | 0.000342 | 0.002138 | 0.001209 | 0.129423 | 0.536435 |
| 68 | F | 39 | 3.45E-05 | 0.000613 | 1.22E-05 | 6.37E-05 | 0.000384 | 0.004011 | 0.120471 |
| 69 | M | 47 | 0.000115 | 0.002603 | 0.000174 | 0.000349 | 0.001525 | 0.061394 | 0.112855 |
| 70 | F | 64 | 0.000138 | 0.001415 | 0.00024 | 0.003109 | 0.001864 | 0.130513 | 0.249192 |
| 71 | M | 62 | 0.000262 | 0.003133 | 0.000284 | 0.000246 | 0.001759 | 0.041246 | 0.401297 |
| 72 | F | 54 | 0.001724 | 0.00558 | 0.000308 | 0.005505 | 0.025207 | 0.910991 | 0.137778 |
| 73 | M | 44 | 7.83E-05 | 0.001386 | 7.99E-05 | 0.000252 | 0.001027 | 0.030608 | 0.270878 |
| 74 | M | 35 | 0.000132 | 0.000922 | 0.000102 | 9.38E-05 | 0.000164 | 0.013729 | 0.002528 |
| 75 | M | 54 | 0.000128 | 0.001558 | 5.74E-05 | 0.000121 | 0.00019 | 0.016646 | 0.001646 |
| 76 | M | 65 | 0.000449 | 0.005201 | 0.000441 | 0.003603 | 0.000283 | 0.073063 | 0.958556 |
| 77 | F | 65 | 0.000457 | 0.002769 | 8.35E-05 | 0.00054 | 0.000142 | 0.020691 | 0.375474 |
| 78 | M | 52 | 6.78E-05 | 0.003333 | 0.00011 | 0.000554 | 0.00163 | 0.008997 | 0.224577 |
| 79 | M | 58 | 0.000566 | 0.00704 | 0.000481 | 0.000666 | 0.001649 | 0.154387 | 0.007418 |
| 80 | F | 45 | 0.00011 | 0.000587 | 3.36E-05 | 8.3E-05 | 7.55E-05 | 0.010188 | 0.001348 |
| 81 | M | 62 | 5.95E-05 | 0.00079 | 3.71E-05 | 6.1E-05 | 5.19E-05 | 0.005787 | 0.000773 |
| 82 | F | 54 | 4.24E-05 | 0.001096 | 4.62E-05 | 4.2E-05 | 4.76E-05 | 0.00693 | 0.000544 |
| 83 | M | 55 | 0.000199 | 0.005741 | 0.000629 | 0.000381 | 0.000246 | 0.121679 | 0.002483 |
| 84 | M | 45 | 0.00012 | 0.000204 | 2.3E-05 | 3.51E-05 | 8.24E-05 | 0.00294 | 0.001707 |
| 85 | M | 56 | 0.000113 | 0.001344 | 4.13E-05 | 0.000271 | 0.001537 | 0.002168 | 0.232874 |
| 86 | F | 56 | 0.000301 | 0.001597 | 4.7E-05 | 0.000425 | 0.001843 | 0.004833 | 0.461175 |
| 87 | F | 52 | 0.001855 | 0.005848 | 0.000223 | 0.001374 | 0.000905 | 0.00911 | 0.635399 |
| 88 | F | 54 | 0.000279 | 0.003095 | 6.27E-05 | 0.000185 | 0.000286 | 0.002293 | 0.311295 |
| 89 | F | 66 | 0.000221 | 0.002084 | 6.38E-05 | 0.000957 | 0.000166 | 0.030418 | 0.208859 |
| 90 | F | 50 | 0.000274 | 0.001539 | 4E-05 | 0.000197 | 0.000168 | 0.010445 | 0.230075 |
| 91 | F | 46 | 0.000299 | 0.004062 | 0.000235 | 0.001036 | 0.000257 | 0.04848 | 0.094703 |
| 92 | F | 57 | 0.00012 | 0.004512 | 0.000161 | 0.000317 | 0.000991 | 0.031184 | 0.068322 |
| 93 | M | 37 | 0.000137 | 0.002735 | 5.36E-05 | 0.000258 | 0.000165 | 0.025088 | 0.75262 |
| 94 | F | 50 | 0.000276 | 0.002718 | 0.000106 | 0.000303 | 0.00013 | 0.027883 | 0.484032 |
| 95 | M | 67 | 0.000377 | 0.003251 | 0.000207 | 0.000712 | 0.000274 | 0.133508 | 0.401297 |
| 96 | M | 35 | 0.000472 | 0.001558 | 0.000115 | 0.000412 | 0.000181 | 0.02314 | 0.080926 |
| 97 | M | 33 | 0.000591 | 0.000905 | 7.79E-05 | 0.000185 | 0.000141 | 0.018617 | 0.081417 |
| 98 | F | 47 | 0.002493 | 0.002477 | 0.00023 | 0.000634 | 0.000446 | 0.028943 | 0.125832 |
| 99 | F | 60 | 0.00046 | 0.00202 | 0.000161 | 0.000703 | 0.000237 | 0.022018 | 0.129537 |
| 100 | M | 40 | 0.000389 | 0.001853 | 8.72E-05 | 0.000622 | 0.000229 | 0.030608 | 0.036872 |
| 101 | M | 47 | 0.003084 | 0.003027 | 0.000269 | 0.037407 | 0.000478 | 0.017514 | 0.286373 |
| 102 | F | 49 | 0.000168 | 0.000434 | 2.36E-05 | 9.27E-05 | 0.000503 | 0.008614 | 0.150677 |
| 103 | M | 56 | 4.93E-05 | 0.000617 | 2.27E-05 | 7.12E-05 | 0.000306 | 0.003148 | 0.22731 |
| 104 | M | 65 | 0.000328 | 0.006377 | 0.140442 | 0.003785 | 0.001616 | 0.063331 | 0.023457 |

| **Gastric cancer** | | | | | | | | | | | |
| --- | --- | --- | --- | --- | --- | --- | --- | --- | --- | --- | --- |
| Number | Age | Gender | Stage | Pathology | miR-17-3p | miR-17-5p | miR-18a-5p | miR-19a-3p | miR-19b-3p | miR-20a-5p | miR-92a-3p |
| 1 | 50 | M | I | adenocarcinoma | 0.001756 | 0.002065 | 0.000141 | 0.00015 | 0.00018171463094+J2:J260177 | 0.001319 | 0.222146 |
| 2 | 56 | M | I | adenocarcinoma | 0.002486 | 0.001469 | 0.000163 | 0.000177 | 0.000152 | 0.002745 | 0.204115 |
| 3 | 60 | M | I | adenocarcinoma | 0.002165 | 0.00213 | 0.000116 | 0.000149 | 0.000119 | 0.004061 | 0.667698 |
| 4 | 68 | F | I | adenocarcinoma | 0.000808 | 0.003212 | 0.000181 | 0.000194 | 1.54E-05 | 0.000787 | 0.149046 |
| 5 | 79 | M | I | adenocarcinoma | 0.009393 | 0.003252 | 0.000171 | 0.000126 | 0.000141 | 0.001364 | 0.577504 |
| 6 | 35 | F | I | adenocarcinoma | 0.002161 | 0.002438 | 0.000125 | 0.000197 | 0.00017 | 0.001447 | 0.168206 |
| 7 | 53 | M | I | adenocarcinoma | 0.002499 | 0.001572 | 0.000109 | 0.000117 | 0.000199 | 0.002797 | 0.115758 |
| 8 | 63 | M | I | adenocarcinoma | 0.001778 | 0.001078 | 0.000148 | 0.000129 | 0.000183 | 0.003349 | 0.059451 |
| 9 | 66 | M | I | adenocarcinoma | 0.000552 | 0.002199 | 0.000222 | 6.15E-05 | 0.000178 | 0.001028 | 0.154552 |
| 10 | 66 | M | I | adenocarcinoma | 0.000981 | 0.001407 | 0.000177 | 1.94E-05 | 0.000145 | 0.0004 | 0.065887 |
| 11 | 67 | F | I | adenocarcinoma | 0.002028 | 0.002025 | 0.000166 | 6.19E-05 | 0.000147 | 0.00307 | 0.296954 |
| 12 | 51 | F | I | adenocarcinoma | 0.012844 | 0.003298 | 0.000112 | 5.89E-05 | 0.000141 | 0.000281 | 0.052046 |
| 13 | 31 | F | I | adenocarcinoma | 2.2E-05 | 0.002169 | 0.000143 | 1.15E-05 | 0.000107 | 0.000552 | 0.035559 |
| 14 | 46 | F | I | adenocarcinoma | 0.002572 | 0.001988 | 0.000113 | 4.05E-05 | 0.000135 | 0.00293 | 0.211656 |
| 15 | 72 | M | I | adenocarcinoma | 0.04661 | 0.002494 | 0.000185 | 5.27E-05 | 0.000129 | 0.005752 | 0.062776 |
| 16 | 55 | M | I | adenocarcinoma | 0.000783 | 0.001534 | 0.000121 | 5.4E-05 | 0.000242 | 0.001493 | 0.062398 |
| 17 | 50 | M | I | adenocarcinoma | 1.79E-05 | 0.000525 | 1.45E-05 | 2.22E-06 | 3.14E-06 | 0.000433 | 0.005013 |
| 18 | 59 | M | I | adenocarcinoma | 0.003325 | 0.002486 | 0.000178 | 1.43E-05 | 0.000127 | 0.060635 | 0.659672 |
| 19 | 71 | M | II | adenocarcinoma | 0.008289 | 0.002472 | 0.000185 | 8.41E-05 | 0.000149 | 0.017495 | 0.151958 |
| 20 | 67 | M | II | adenocarcinoma | 0.04603 | 0.000572 | 0.00015 | 0.000156 | 0.000144 | 0.008095 | 0.171492 |
| 21 | 33 | F | II | adenocarcinoma | 0.000414 | 0.002416 | 0.000144 | 2.67E-05 | 0.000141 | 0.001438 | 0.300567 |
| 22 | 66 | M | II | adenocarcinoma | 0.001098 | 0.001228 | 0.000141 | 0.000132 | 0.000126 | 0.001122 | 0.442597 |
| 23 | 52 | M | II | adenocarcinoma | 0.001846 | 0.001746 | 0.000102 | 0.000229 | 0.000174 | 0.040484 | 0.161235 |
| 24 | 61 | M | II | adenocarcinoma | 0.017672 | 0.001683 | 0.000128 | 9.93E-05 | 0.000147 | 0.00127 | 0.04289 |
| 25 | 66 | M | II | adenocarcinoma | 0.066584 | 0.001683 | 0.000125 | 5.02E-05 | 0.00015 | 0.001456 | 0.063157 |
| 26 | 75 | M | II | adenocarcinoma | 0.000207 | 0.002374 | 0.000142 | 4.23E-05 | 0.00017 | 0.01461 | 0.104956 |
| 27 | 67 | M | II | adenocarcinoma | 0.000228 | 0.002377 | 0.000176 | 0.000521 | 0.000142 | 3.78E-06 | 0.021013 |
| 28 | 59 | M | II | adenocarcinoma | 0.000545 | 0.002402 | 0.000203 | 1.4E-05 | 0.000121 | 0.000163 | 0.194476 |
| 29 | 60 | M | III | adenocarcinoma | 0.007884 | 0.00114 | 0.000149 | 3.19E-05 | 0.000142 | 0.000954 | 0.237424 |
| 30 | 22 | M | III | adenocarcinoma | 0.006373 | 0.006237 | 0.000161 | 0.000185 | 0.00023 | 0.006513 | 0.170252 |
| 31 | 46 | M | III | adenocarcinoma | 0.000871 | 0.002427 | 0.000131 | 6.78E-05 | 0.000156 | 0.001926 | 0.104324 |
| 32 | 46 | M | III | adenocarcinoma | 0.001835 | 0.002344 | 0.000193 | 0.00023 | 0.000103 | 0.012585 | 0.897959 |
| 33 | 67 | M | III | adenocarcinoma | 0.001639 | 0.001966 | 0.000229 | 1.6E-05 | 5.57E-05 | 0.000176 | 0.015251 |
| 34 | 64 | M | III | adenocarcinoma | 0.002492 | 0.001816 | 0.000174 | 0.000143 | 0.000111 | 0.002976 | 0.041865 |
| 35 | 64 | F | III | adenocarcinoma | 0.00043 | 0.001779 | 0.000148 | 9.16E-06 | 0.000117 | 0.001016 | 0.139455 |
| 36 | 80 | M | III | adenocarcinoma | 0.002473 | 0.002414 | 0.000164 | 6.19E-05 | 0.000299 | 0.027711 | 0.241771 |
| 37 | 58 | M | III | adenocarcinoma | 0.002172 | 0.002354 | 0.000179 | 0.000325 | 0.000177 | 0.002063 | 0.149046 |
| 38 | 71 | M | III | adenocarcinoma | 0.00058 | 0.001901 | 0.000129 | 8.31E-05 | 1.63E-05 | 2.35E-05 | 0.085452 |
| 39 | 59 | M | III | adenocarcinoma | 0.000168 | 0.000756 | 3.98E-05 | 1.78E-05 | 2.86E-06 | 2.25E-05 | 0.011409 |
| 40 | 58 | M | III | adenocarcinoma | 0.000452 | 0.001243 | 0.0002 | 4.66E-05 | 4.63E-05 | 2.55E-05 | 0.05803 |
| 41 | 35 | M | III | adenocarcinoma | 9.05E-05 | 0.000276 | 1.41E-05 | 1.41E-06 | 2.84E-06 | 0.002967 | 0.002516 |
| 42 | 43 | M | III | adenocarcinoma | 1.46E-05 | 0.000393 | 7.03E-05 | 1.87E-06 | 3.19E-06 | 5.21E-05 | 0.011478 |
| 43 | 69 | F | III | adenocarcinoma | 1.71E-05 | 0.000266 | 7.35E-05 | 1.31E-06 | 1.92E-06 | 1.87E-05 | 0.030942 |
| 44 | 71 | F | III | adenocarcinoma | 0.000666 | 0.001303 | 7.7E-05 | 1.89E-06 | 0.000105 | 0.004321 | 0.068736 |
| 45 | 45 | F | III | adenocarcinoma | 0.000108 | 0.001166 | 1.06E-05 | 2.52E-06 | 1.11E-05 | 0.001926 | 0.06354 |
| 46 | 67 | M | III | adenocarcinoma | 0.000409 | 0.003818 | 2.63E-05 | 6.07E-06 | 2.38E-05 | 0.00568 | 0.09244 |
| 47 | 34 | F | IV | adenocarcinoma | 0.001214 | 0.006826 | 0.000134 | 7.96E-05 | 0.000228 | 0.015938 | 0.253753 |
| 48 | 72 | M | IV | adenocarcinoma | 0.001447 | 0.001642 | 0.000143 | 4.15E-05 | 0.000129 | 0.001777 | 0.416627 |
| 49 | 69 | F | IV | adenocarcinoma | 0.001325 | 0.00691 | 0.000186 | 2.45E-05 | 6.08E-05 | 0.002222 | 0.053969 |
| 50 | 56 | M | IV | adenocarcinoma | 0.000457 | 0.008474 | 0.000141 | 0.00014 | 0.000127 | 0.002012 | 0.14995 |
| 51 | 56 | M | IV | adenocarcinoma | 0.002162 | 0.027929 | 0.000137 | 5.47E-05 | 0.000157 | 0.002728 | 0.122084 |
| 52 | 57 | F | IV | adenocarcinoma | 0.003143 | 0.002462 | 9.34E-05 | 4.55E-05 | 0.000143 | 0.004189 | 0.040864 |
| 53 | 53 | M | IV | adenocarcinoma | 0.005152 | 0.002118 | 0.000195 | 0.000317 | 0.00074 | 0.002307 | 0.445281 |
| 54 | 51 | M | IV | adenocarcinoma | 0.00022 | 0.008686 | 3.27E-05 | 2.01E-05 | 0.000108 | 0.000328 | 0.03307 |
| 55 | 61 | M | IV | adenocarcinoma | 0.000335 | 0.002989 | 1.96E-05 | 7.3E-06 | 0.000118 | 0.000563 | 0.079953 |
| 56 | 66 | M | IV | adenocarcinoma | 0.001058 | 0.00316 | 5.21E-05 | 1.78E-05 | 0.000174 | 0.001403 | 0.070419 |
| 57 | 53 | M | IV | adenocarcinoma | 4.72E-05 | 0.001355 | 0.000135 | 6.91E-05 | 0.000299 | 0.001101 | 0.162212 |
| 58 | 56 | M | IV | adenocarcinoma | 0.018931 | 0.003576 | 0.000121 | 2.94E-05 | 0.000282 | 0.053548 | 0.09244 |
| 59 | 20 | F | IV | adenocarcinoma | 0.002605 | 0.00201 | 0.000165 | 2.42E-05 | 0.00017 | 0.004893 | 1.136769 |
| 60 | 58 | M | IV | adenocarcinoma | 0.000741 | 0.001662 | 0.000121 | 1.09E-05 | 8.34E-05 | 0.000351 | 0.062398 |
| 61 | 67 | M | IV | adenocarcinoma | 0.001966 | 0.002269 | 0.000185 | 0.000337 | 0.002223 | 0.008776 | 1.019538 |
| 62 | 67 | M | IV | adenocarcinoma | 0.002302 | 0.002609 | 0.000136 | 0.000116 | 0.000151 | 0.001386 | 0.311672 |
| 63 | 65 | F | IV | adenocarcinoma | 0.002861 | 0.006299 | 0.000209 | 0.000405 | 0.00015 | 0.001951 | 0.079471 |
| 64 | 60 | M | IV | adenocarcinoma | 0.016809 | 0.003257 | 0.000102 | 7.91E-05 | 0.000157 | 0.016543 | 0.304224 |
| 65 | 66 | M | IV | adenocarcinoma | 0.005588 | 0.001576 | 0.000133 | 0.000186 | 0.000166 | 0.003412 | 0.173369 |
| 66 | 74 | F | IV | adenocarcinoma | 0.003143 | 0.001478 | 0.000121 | 2.91E-05 | 0.000189 | 0.007751 | 0.067094 |
| 67 | 65 | M | IV | adenocarcinoma | 0.000595 | 0.001386 | 0.000151 | 4.18E-05 | 0.00013 | 0.02832 | 0.175478 |
| 68 | 61 | F | IV | adenocarcinoma | 0.001953 | 0.002011 | 0.001652 | 6.91E-05 | 0.000239 | 0.00143 | 0.02076 |
| 69 | 55 | M | IV | adenocarcinoma | 0.000367 | 0.001699 | 0.00012 | 1.44E-05 | 9.32E-07 | 0.000102 | 0.033472 |
| 70 | 73 | M | IV | adenocarcinoma | 0.000128 | 0.000899 | 0.000331 | 2.36E-05 | 4.8E-06 | 1.64E-05 | 0.03113 |
| 71 | 59 | M | IV | adenocarcinoma | 0.000691 | 0.001309 | 0.011832 | 0.000354 | 2.13E-05 | 0.008997 | 0.185292 |
| 72 | 59 | M | IV | adenocarcinoma | 6.83E-06 | 0.000283 | 8.02E-06 | 1.29E-06 | 2.47E-07 | 2.83E-05 | 0.003244 |
| 73 | 64 | M | IV | adenocarcinoma | 1.75E-05 | 0.000153 | 5.48E-06 | 8.04E-06 | 1.8E-05 | 5.14E-05 | 0.002369 |
| 74 | 55 | M | IV | adenocarcinoma | 3.74E-05 | 0.000467 | 2.02E-05 | 2.61E-06 | 7.82E-06 | 0.024778 | 0.004258 |
| 75 | 45 | M | IV | adenocarcinoma | 0.00046 | 0.002533 | 0.000117 | 9.1E-06 | 8.44E-05 | 6.27E-05 | 0.21294 |

| **Healthy control** | | | | | | | | | |
| --- | --- | --- | --- | --- | --- | --- | --- | --- | --- |
| Number | Age | Gender | miR-17-3p | miR-17-5p | miR-18a-5p | miR-19a-3p | miR-19b-3p | miR-20a-5p | miR-92a-3p |
| 1 | 24 | M | 0 | 0.0037947 | 1.431E-05 | 5.669E-05 | 9.942E-05 | 4.538E-05 | 0.0522983 |
| 2 | 23 | F | 0 | 0.0011163 | 8.995E-05 | 5.846E-05 | 0.0001264 | 0.0001416 | 0.0738197 |
| 3 | 22 | F | 0 | 0.0003739 | 3.69E-05 | 9.914E-05 | 1.865E-05 | 5.305E-05 | 0.2371374 |
| 4 | 24 | F | 0 | 0.0006642 | 0.0001472 | 7.997E-05 | 2.809E-05 | 0.0018675 | 0.1861907 |
| 5 | 40 | M | 0 | 0.0005124 | 6.074E-05 | 0.0002851 | 4.085E-05 | 0.0001665 | 0.7995325 |
| 6 | 25 | M | 0 | 0.0008145 | 3.878E-05 | 0.0001744 | 5.284E-05 | 4.627E-05 | 0.0400812 |
| 7 | 26 | M | 0 | 0.0002426 | 1.714E-05 | 5.846E-05 | 1.175E-05 | 4.955E-05 | 0.0658078 |
| 8 | 25 | F | 0 | 7.406E-05 | 2.976E-06 | 1.107E-05 | 5.087E-06 | 0.0001739 | 0.0168817 |
| 9 | 25 | F | 0 | 0.0001034 | 1.741E-06 | 6.566E-06 | 5.958E-06 | 4.59E-05 | 0.006857 |
| 10 | 24 | M | 0 | 0.0001357 | 1.96E-06 | 1.184E-05 | 8.415E-06 | 0.0001452 | 0.0159875 |
| 11 | 25 | M | 0 | 0.000186 | 2.899E-06 | 1.571E-05 | 9.404E-06 | 0.0003118 | 0.1444321 |
| 12 | 34 | M | 0 | 0.0001738 | 2.325E-06 | 1.441E-05 | 8.974E-06 | 0.0002161 | 0.0196366 |
| 13 | 32 | M | 0 | 0.0001594 | 4.245E-06 | 1.389E-05 | 7.707E-06 | 0.0001282 | 0.007976 |
| 14 | 35 | M | 0 | 0.0002471 | 2.055E-06 | 1.61E-05 | 6.207E-06 | 0.0004788 | 0.0558951 |
| 15 | 34 | M | 0 | 0.0001623 | 2.833E-06 | 1.754E-05 | 1.565E-05 | 5.371E-05 | 0.1335135 |
| 16 | 40 | M | 0 | 0.0022861 | 3.667E-05 | 2.938E-05 | 2.012E-05 | 0.0004556 | 0.2259392 |
| 17 | 28 | M | 0 | 0.0002938 | 9.251E-06 | 1.888E-05 | 7.618E-06 | 0.0001071 | 0.0099758 |
| 18 | 43 | F | 0 | 0.0009447 | 2.213E-05 | 2.615E-05 | 2.776E-05 | 0.00045 | 0.1954188 |
| 19 | 28 | M | 0 | 0.0015489 | 4.675E-05 | 2.083E-05 | 9.712E-05 | 0.00039 | 0.0133352 |
| 20 | 40 | M | 0 | 0.0006425 | 5.383E-06 | 5.618E-05 | 1.765E-05 | 0.0003919 | 0.1986107 |
| 21 | 37 | M | 0 | 0.0005392 | 3.757E-05 | 1.818E-05 | 7.768E-06 | 0.0005519 | 0.0528923 |
| 22 | 32 | M | 0 | 0.0003858 | 3.869E-05 | 1.61E-05 | 8.749E-06 | 0.0006588 | 0.1531535 |
| 23 | 36 | F | 0 | 0.0002809 | 0.0001674 | 1.107E-05 | 8.571E-06 | 6.492E-05 | 0.2741347 |
| 24 | 27 | F | 0 | 0.0002215 | 1.872E-06 | 2.938E-05 | 2.921E-05 | 5.855E-05 | 0.013759 |
| 25 | 41 | M | 0 | 0.0002582 | 8.85E-05 | 0.0001744 | 0.0001264 | 0.0004975 | 0.1841958 |
| 26 | 44 | M | 0 | 0.0001796 | 6.27E-05 | 5.846E-05 | 1.175E-05 | 0.0001238 | 0.0164637 |
| 27 | 38 | M | 0 | 0.0012163 | 2.341E-05 | 1.754E-05 | 9.712E-05 | 0.0001684 | 0.0670897 |
| 28 | 37 | M | 0 | 0.0001594 | 4.492E-06 | 1.571E-05 | 5.958E-06 | 4.622E-05 | 0.0077911 |
| 29 | 43 | M | 0 | 0.0017459 | 9.105E-06 | 0.0002851 | 9.404E-06 | 0.0004891 | 0.0098759 |
| 30 | 33 | M | 0 | 7.655E-05 | 1.321E-05 | 6.566E-06 | 4.085E-05 | 0.000396 | 0.7952359 |
| 31 | 42 | M | 0 | 0.0001301 | 4.251E-06 | 2.083E-05 | 6.207E-06 | 5.513E-05 | 0.0531599 |
| 32 | 26 | F | 0 | 0.0001537 | 6.351E-06 | 1.441E-05 | 5.284E-05 | 0.0001058 | 0.143231 |
| 33 | 23 | F | 0 | 0.0009348 | 4.765E-05 | 1.389E-05 | 7.707E-06 | 0.0002461 | 0.2235932 |
| 34 | 32 | F | 0 | 0.0001574 | 1.642E-06 | 7.997E-05 | 5.087E-06 | 0.0014875 | 0.0414208 |
| 35 | 27 | M | 0 | 0.0036784 | 3.782E-05 | 9.914E-05 | 2.776E-05 | 0.0001565 | 0.0133352 |
| 36 | 38 | F | 0 | 0.0021861 | 2.857E-06 | 5.669E-05 | 9.942E-05 | 0.0001264 | 0.015682 |
| 37 | 36 | F | 0 | 0.0001623 | 4.692E-06 | 1.184E-05 | 1.565E-05 | 4.729E-05 | 0.0718397 |
| 38 | 29 | M | 0 | 0.0008145 | 1.615E-05 | 2.615E-05 | 2.012E-05 | 4.759E-05 | 0.0067505 |
